# Systemic Risk Analysis on Reconstructed Economic and Financial Networks

**DOI:** 10.1038/srep15758

**Published:** 2015-10-28

**Authors:** Giulio Cimini, Tiziano Squartini, Diego Garlaschelli, Andrea Gabrielli

**Affiliations:** 1 Istituto dei Sistemi Complessi (ISC-CNR) UoS “Sapienza” Università di Roma, 00185 Rome, Italy; 2 Lorentz Institute for Theoretical Physics, University of Leiden, 9506 Leiden, Netherlands; 3 IMT Institute for Advanced Studies, 55100 Lucca, Italy

## Abstract

We address a fundamental problem that is systematically encountered when modeling real-world complex systems of societal relevance: the limitedness of the information available. In the case of economic and financial networks, privacy issues severely limit the information that can be accessed and, as a consequence, the possibility of correctly estimating the resilience of these systems to events such as financial shocks, crises and cascade failures. Here we present an innovative method to reconstruct the structure of such partially-accessible systems, based on the knowledge of intrinsic node-specific properties and of the number of connections of only a limited subset of nodes. This information is used to calibrate an inference procedure based on fundamental concepts derived from statistical physics, which allows to generate ensembles of directed weighted networks intended to represent the real system—so that the real network properties can be estimated as their average values within the ensemble. We test the method both on synthetic and empirical networks, focusing on the properties that are commonly used to measure systemic risk. Indeed, the method shows a remarkable robustness with respect to the limitedness of the information available, thus representing a valuable tool for gaining insights on privacy-protected economic and financial systems.

The estimation of the structural properties of a complex network when the available information on the system is incomplete represents an unsolved challenge^1^,^2^, yet it brings to many important applications. The most typical case is that of financial networks, whose nodes represent financial institutions and links stand for financial ties (*e.g*., loans or derivative contracts)—the latter indicating dependencies among the institutions themselves, allowing for the propagation of financial distress across the network. The resilience of the system to the default or the distress of one or more institutions considerably depends on the topology of the whole network^3^,^4^,^5^; however, because of confidentiality issues, the information on mutual exposures that regulators are able to collect is very limited^6^. Systemic risk analysis has been typically pursued by reconstructing the unknown links of the network using maximum entropy approaches^7^,^8^,^9^. These methods are also known as “dense reconstruction” techniques because they assume that the network is fully connected—an hypothesis that represents their strongest limitation. In fact, not only real networks show a largely heterogeneous distribution of the connectivity, but such a dense reconstruction was shown to lead to systemic risk underestimation^2^,^9^. More refined techniques like “sparse reconstruction” algorithms^2^ allow to obtain a network with arbitrary density, however they still underestimate systemic risk because of the homogeneity principle used to assign link weights. A more recent approach^10^,^11^, which builds on even earlier results^12^, instead uses the limited topological information on the network to generate an ensemble of graphs using the *configuration model* (CM)^13^—where, however, the Lagrange multipliers that define it are replaced by *fitnesses*, *i.e*. known intrinsic node-specific features^14^. The average values of the observables computed on the CM-induced ensemble are then used as estimates for the real network properties. The latter approach overcomes the heterogeneity issue described above, yet it only allows to reconstruct systems in which each tie is undirected and unweighted—thus limiting the analysis to unrealistic and oversimplified configurations. Indeed, link directionality has been shown to play an important role in contagion processes and percolation analysis over these and other systems^15^,^16^ by, *e.g*., speeding up or confining the infection with respect to the undirected case. Since real economic and financial networks are, by their nature, directed, links directionality has to be taken into account when assessing their robustness to shock and crashes. Moreover, the connection weights between the entities of these systems often assume heterogeneous values, which in turn strongly affect the way such entities react to the default or distress of their interacting partners^4^. A recent study^17^ has shown that, in order to satisfactorily reconstruct weighted networks, the procedure described above^10^,^11^ cannot be extended naively by enforcing the corresponding weighted information, otherwise the reconstructed network is unrealistically dense^18^. Rather, one should employ a nontrivial combination of weighted and binary properties^17^. However, while this approach is feasible when such properties can be empirically accessed^17^,^19^, it cannot be used when the system is privacy-protected (as in interbank and other financial networks).

In order to achieve a realistic and faithful reconstruction of economic and financial networks, here we develop an improved procedure that allows to reconstruct links directionality, and at the same time we implement an effective and self-consistent prescription to assign link weights. Our method can thus be employed specifically for systemic risk estimation, by assessing those network properties that have been shown to play a crucial role in contagion processes and in the propagation of distress over a networked system: the k-core structure^20^, the percolation threshold^21^, the mean shortest path length^22^ and the DebtRank^4^. In particular, we perform an extensive analysis in order to quantify the accuracy of our method with respect to the size of the subset of nodes for which the topological information is available. Validation of the method is carried out on benchmark synthetic networks generated through a fitness-induced CM, as well as on two representative empirical systems, namely the International Trade Network or World Trade Web (WTW)^23^ and the Electronic Market for Interbank Deposits (E-mid)^24^. In both cases, we have full information on these systems and we can thus unambiguously assess the accuracy of the method in reconstructing them.

## Previous approaches

Before explaining our method in detail, let us introduce some notation and recall previous results that we build upon. We will deal with weighted directed networks, *i.e*., graphs composed by a set *V* of nodes (with 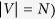 and described by a weighted directed adjacency matrix—whose generic element 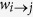 represents the weight of the connection that runs from node *i* to node *j*. The incoming total weight or *in-strength* for a generic node *i* is then defined by 
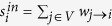
, whereas, its outgoing total weight or *out-strength* reads 
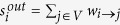
. It is also convenient to introduce the binary directed adjacency matrix that describes the binary topology: 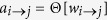 (Θ is the Heaviside step function: 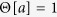 for *a* > 0 and 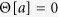 otherwise). This allows to define node *i*’s number of incoming connections or *in-degree* 
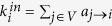
 and number of outgoing connections or *out-degree* 
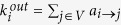
. Finally, the binary undirected adjacency matrix—whose elements are obtained as 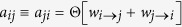
—is used to define the number of incident connections or undirected *degree* of node *i*: 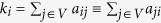.

In what follows, we are going to suppose that we only have partial information about the network: rather than knowing all the entries of the weighted adjacency matrix, we assume to know only local, node-specific information. In general, this information can be either topological (*e.g*., the degrees of nodes^18^) or non-topological (*e.g.*, the economic size of nodes^12^). Before describing our specific implementation, we recall some important results that have been found previously using both schemes. At a binary network level, it has been shown that the topology of economic networks (including the ones we consider in this paper) can be accurately reconstructed from the knowledge of node degrees only^18^,^25^,^26^. Alternatively, since node degrees often turn out to be in an approximately monotonic (but highly nonlinear) relationship with some intrinsic economic property of nodes (like the GDP of countries in the WTW^12^ or the portfolio volume in case of shareholding networks^27^), a good binary network reconstruction can be also achieved starting from the knowledge of such intrinsic node properties, rather than from node degrees themselves. The earliest and most clearcut illustration of this nontrivial result has been provided for the WTW^12^,^28^, where it was shown that the observed topology can be reproduced from the knowledge of the GDP of all countries, plus the total number of links. This result, which supports the hypotheses of the *fitness model*^14^, was later shown to remain valid even if one assumes to know the degrees of only a small subset of the nodes^10^,^29^ (a framework known as *bootstrap* that we use also below), and if the analysis is extended to other financial systems such as interbank networks. The robustness of the reconstruction under bootstrap for interbank networks is very important for concrete applications, since knowing even only the total number of interbank connections is practically impossible, while knowing the degree of a few banks is in many cases easier^30^. Using the above technique, the level of systemic risk associated with the *binary* structure of a financial network can be estimated fairly well^10^,^29^.

On the other hand, at a weighted network level the situation is much more complicated, and still unsolved at present. If one attempts to reconstruct the network starting from the strengths of nodes (the most direct proxy for nodes size) and without adjusting manually the network density^31^,^32^,^33^, the result is—depending on the methodology adopted—either a very dense network^18^, or a completely connected one^6^. Indeed, in the latter case the link weights are assigned according to the so-called “gravity model” as:


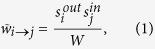


where 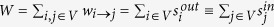 is the total observed weight^6^. The above formula shows that the reconstructed in-strength and out-strength of each node *i*, which are given by 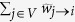 and 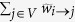 respectively, coincide with the observed quantities 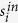 and 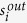 as desired. However, it also highlights that the reconstructed network is fully connected, a limitation that can be understood as the result of the fact that, in absence of purely topological information, the known total weight is redistributed over many more (all, in fact) pairs of nodes than those actually connected in the real network^17^. As we have mentioned in the Introduction, in the case of interbank networks this results in a very poor estimation of systemic risk.

Recently it has been shown that, in order to satisfactorily reconstruct a weighted network, one should simultaneously specify both node strengths and node degrees^17^. This results in an accurate reconstruction, however requires the knowledge of a lot of information. How to relax this requirement in an effective manner is an open question at the moment. For the WTW, a recent study^19^ has shown that, as in the purely binary case^12^, it is possible to reproduce both the topology and the weighted structure of the network by replacing the knowledge of the degree and strength sequence with that of the total number of links and total link weight respectively, plus the knowledge of the GDP of all countries. While powerful, this simplification is generally not feasible for financial networks^34^. In particular, for real interbank networks the full strength sequences (*i.e*., total loans and liabilities) are typically publicly available—thus there is no need to assume that it is unknown, whereas, the total number of links is not (since, as we have already mentioned, it is feasible to collect information on the connectivity for only a subset of nodes). The aim of this paper is to introduce a reconstruction method that is appropriate for directed and weighted financial networks, and that allows to estimate systemic risk to a high level of accuracy.

## Method

In accordance with the above discussion, in this paper we are going to adopt a bootstrap-like scenario and assume incomplete information about the topology of a given network *G*_0_. In particular, we suppose to know the in-degree and out-degree sequences 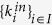 and 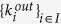 only for a subset 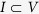 of all nodes (where 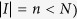. Moreover, we suppose to know a pair of properties 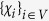 and 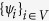 for *all* the nodes—that will be our *fitnesses*. These fitnesses should be thought of as intrinsic economic properties that are responsible for the inward (in-degree) and outward (out-degree) connectivity of nodes (see points I and II below); in this respect, it is quite straightforward (and actually very common^2^,^6^,^7^,^8^,^9^,^17^,^18^,^31^,^32^,^33^) to associate them with the nodes in- and out- strengths, respectively—but in general other proxies can be used. Given these ingredients, our network reconstruction method invokes a two-step statistical procedure (in which connection probabilities are estimated first, and link weights later) in order to find the most probable estimate for the value 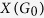 of a given property *X* computed on the network *G*_0_, compatible with the constraints given by the aforementioned information we have on *G*_0_.

First, we aim at reconstructing the binary topology of the network. To this end, we build on two important hypotheses.

I) The binary topology of *G*_0_ is drawn from an ensemble Ω induced by a directed CM^25^—meaning that Ω is a set of binary directed networks that are maximally random, except for the ensemble averages (*i.e*., expected values) of the in- and out- degrees 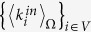 and 
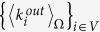
 that are constrained to the observed values 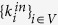 and 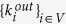, respectively^13^. The directed CM prescribes that the probability distribution over Ω is defined via a set of Lagrange multipliers 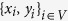 (two for each node), whose values can be adjusted in order to satisfy the equivalence 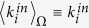 and 
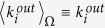
, 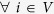
25. The values of *x*_*i*_ and *y*_*i*_ are thus induced by the in- and out- degree of node *i*, respectively, and the ensemble probability for a directed connection between any two nodes *i* and *j* reads^13^:


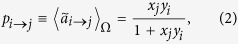


where 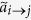 is a particular realization of the reconstructed link, having expected value over the ensemble equal to 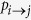: 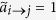 with probability 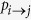, and 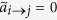 otherwise. Eq. (2) thus shows that *x*_*i*_ (*y*_*i*_) quantifies the ability of node *i* to receive incoming (form outgoing) connections.

II) The fitnesses 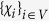 and 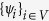 are assumed to be linearly correlated, respectively, to the in-degree-induced and out-degree-induced Lagrange multipliers 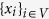 and 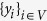 through universal (unknown) parameters *α* and *β*: 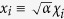 and 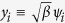, 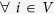. Therefore eq. (2) becomes:


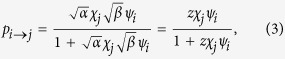


where we have defined 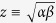. Such an hypothesis is inspired by the *fitness model*^14^, which assumes the network topology to be determined by intrinsic properties associated to each node of the network. We recall that this approach has been already used in the past to model several economic and financial networks^12^,^24^, possibly within the CM framework assuming a connection between fitnesses and Lagrange multipliers^27^.

These two hypotheses allow us to build the optimal CM ensemble Ω induced by the fitnesses 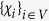 and 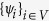, that is compatible with the binary constraints on *G*_0_—given by the knowledge of 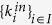 and 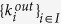. Indeed, because of the limited available information, finding the CM of the real system^13^ is impossible, and we thus have to impose it by assigning *ad hoc* values to the Lagrange multipliers—whence the name “fitness-induced” CM (FiCM). In practice, since we know the fitness values 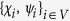, in order to determine unambiguously Ω we have to find the most likely value of the proportionality constant *z* that defines Ω according to eq. (3). This can be done using the partial knowledge of the degree sequences to estimate the appropriate value of *z* through a maximum-likelihood argument^12^, *i.e*., by comparing, for the nodes in the set *I*, the average number of incoming and outgoing connections in the ensemble Ω with their in-degrees and out-degrees observed in *G*_0_:


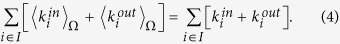


In the above expression, 
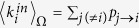
 and 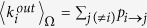 contain the unknown parameter *z* through eq. (3), and since 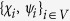 and 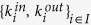 are known, eq. (4) defines an algebraic equation in *z*, whose solution allows to build the FiCM ensemble—even with the knowledge of the in- and out- degree of just a single node.

We now turn to reconstructing the weighted topology of *G*_0_. A key ingredient of our approach will be the following consideration. As already mentioned, eq. (1) ensures that the reconstructed in- and out-strengths of all nodes are equal to the observed ones *only when the reconstructed network is fully connected*. However, if the topology is more complex (hence determined by a nontrivial probability 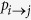 that node *i* connects to node *j*), then in order to reproduce the observed strengths eq. (1) has to be modified as follows:


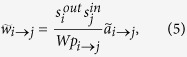


This prescription ensures that the expected value of the reconstructed in-strength and out-strength of node *i* are


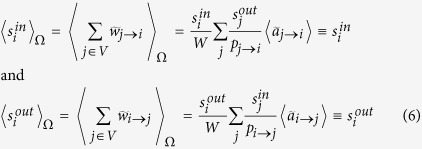


as desired: eq. (5) ensures that the observed in- and out-strength sequences are correctly replicated by the method, irrespectively of whether the topology (as predicted by the set 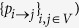 is reproduced. For instance, if 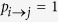 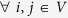 we recover the standard eq. (1)^6–9^, whereas, if 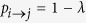 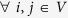 we recover a variant of the sparse reconstruction method^2^. Our purpose here is ensuring that 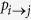 correctly reconstructs the degree sequence, and hence both the binary and weighted topology of the network.

**Figure 1:**
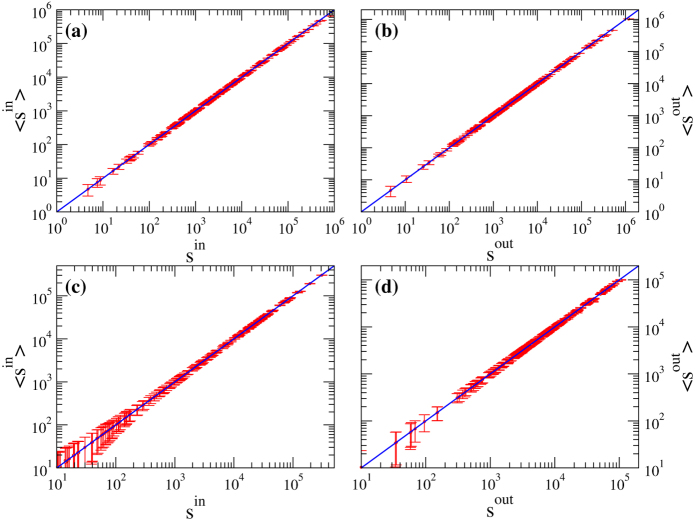
Reconstruction of the strength sequences. Scatter plots of node in-strengths *s*^*in*^ and out-strengths *s*^*out*^ observed for the real network *G*_0_ and their ensemble averages obtained from eq. (8). Upper panels (**a**,**b**) refer to WTW, lower panels (**c**,**d**) to E-mid.

We formalize the above discussion as follows. In the most general case (*i.e*., for generic node fitnesses), in order to obtain a weighted topology we place 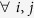 a weight 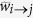 on the directed link from *i* to *j* according to the following prescription:


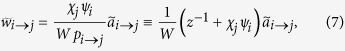


where the last equality comes from eq. (3). In this expression, the normalization *W* represents the expected *induced total weight of the network*, defined as the geometric mean of the sum of the fitnesses: 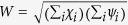. Indeed, this definition is consistent with 
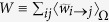
, where 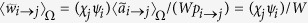. This procedure assures that the expected values of a node *i*’s total in- and out-strengths are directly proportional to 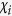 and 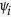, respectively and 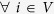. Now, using the natural interpretation of fitnesses as the empirical nodes strengths observed in *G*_0_ 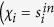 and 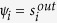, 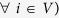, brings to the situation described in the previous paragraph: 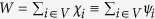, 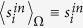 and 
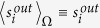
. We stress again that in this way we successfully preserve, on average, the strength sequences of the real network *G*_0_ (and thus its total weight), as shown in Fig. 1. In other words, our network reconstruction method is based on a null model constraining the in-degree and out-degree sequence of a subset of nodes, together with the in-strength and out-strength sequence of the whole set of nodes. The final result is that the appropriate modification of the standard gravity model of eq. (1) is, as for eq. (5), the “degree-corrected gravity model”:


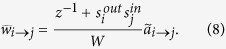


**Figure 2:**
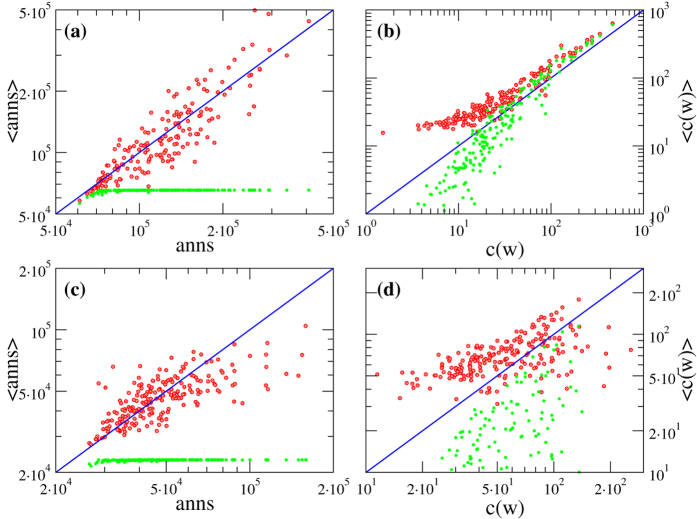
Reconstruction of two higher order properties of directed weighted networks: the average nearest neighbor strength *anns* [panels (a,c)] and the weighted clustering coefficient *c*(*w*) [panels (b,d)] (refer to^37^ for their formal definition). Scatter plots of these quantities observed for the real network *G*_0_ and their ensemble averages obtained from the degree-corrected gravity model of eq. (8) (red circles) or from the standard gravity model of eq. (1) (green asterisks). Upper panels (**a**,**b**) refer to WTW, lower panels (**c**,**d**) to E-mid. Remarkably, our degree-corrected gravity allows to obtain fairly accurate estimates for the *anns*, whereas, the standard gravity model completely fails in this respect as the resulting reconstructed network is fully connected. The degree-corrected gravity model outperforms the standard gravity model also in the reconstruction of *c*(*w*). In this latter case, note that 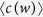 systematically overestimates the real *c*(*w*), because in the definition of this quantity the number of reciprocal links plays an important role, yet it is slightly underestimated by the method (see Figs 5 and 6, and refer to the discussion in^26^).

With respect to eq. (1), eq. (8) has two important differences. On one hand, only the links that are actually created are assigned a non-zero weight; on the other hand, with respect to eq. (1) there is an extra offset *z*^−1^ which depends (through 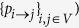 on the observed density, and whose role is precisely that of redistributing the “missing” weight (required to reconstruct the desired in- and out-strengths) from the disconnected pairs of nodes to the connected ones. Remarkably, these modifications also allow to obtain much better estimates of higher order weighted network properties, as compared to the standard gravity approach (Fig. 2).

Finally, once the FiCM ensemble Ω is determined and link weights are placed, statistical mechanics of networks prescribes that the value 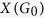 of property *X* computed on *G*_0_ typically varies in the range 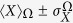, where 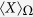 and 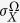 are respectively average and standard deviation of *X* estimated over Ω^13^. We can thus use 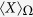 as a good estimation for 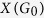.

Summing up, the algorithm works as follows. Given a network *G*_0_, two fitness values *χ* and *ψ* for each of the *N* nodes, and the in-degrees and out-degrees only for a subset *I* of 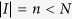 nodes:

- We compute the sum of the in-degrees and out-degrees of the nodes in *I*, and use it to obtain the value of *z* by solving eq. (4);
- Using such *z*, we generate the FiCM ensemble Ω by placing a directed link from any node *i* to any node *j* with probability 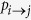 of eq. (3), and assigning it with the corresponding weight 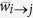 of eq. (7)—provided its existence;
- We compute the estimate of 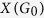 as 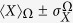 in the FiCM ensemble, typically numerically (*i.e*., by measuring it on networks drawn from Ω).

### Empirical Dataset

In order to test our network reconstruction method, we use two representative empirical systems of economic and financial nature. The first one is the international trade network of the World Trade Web (WTW)^23^, *i.e*., the network whose nodes are the countries and links represent trade volumes between them: thus, 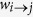 is the monetary flux from country *i* to country *j* (the “amount” of the export from *j* to *i*). The second one is the (E-mid) Electronic Market for Interbank Deposits^24^: in this case, the nodes are banks and a link 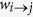 from bank *i* to bank *j* represents the amount of the loan that *i* granted to *j*.

In the following analysis we will use and show results for WTW trade volume data of year 2000, and E-mid aggregated transaction data of year 1999 (both temporal snapshots correspond to the largest size of the network). Analyses for other annual snapshots are reported in the Supplementary Information, and bring to comparable results. In the light of the above observations, we will use as fitnesses 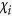 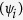 the real node in-strength 
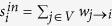
 (out-strength 
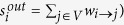
, *i.e*., the total import (export) volumes of countries for WTW, and with the total liquidity borrowed (lent) by banks for E-mid. Note that the goodness of any choice for the fitness values must be first validated according to hypothesis II of our method (as discussed in the first part of section Results).

### Topological Properties

As stated in the Introduction, we will test our network reconstruction method focusing on the network properties (each playing the role of *X* in the discussion of section Methods) which are commonly regarded as the most significant for describing the network resilience to systemic shocks and crashes. We first consider two properties defined for undirected networks (in order to reconstruct these properties, we use the undirected version of the method^10^):

- Degree of the main core *k*^*main*^ and size of the main core *S*^*main*^, where a *k*-core is defined as the “largest connected subgraph whose nodes all have at least *k* connections” (within this subgraph), and the main core is the *k*-core with the highest possible degree (*k*^*main*^)^35^. The main core is relevant to our analysis as it consists of the most influential spreaders (of, *e.g*., an infection or a shock) in a network^20^.
- Size of the giant component *S*_*GC*_ at the bond percolation threshold 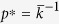 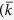 is the mean degree of the network), where bond percolation is the process of occupying each link of the network with probability *p*, and p* is the critical value of *p* at which a percolation cluster containing a finite fraction of all nodes first occurs^21^. Note that the percolation threshold at 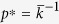 (that we take as reference value) is a feature proper of homogeneous graphs in the infinite volume limit, whereas, for scale-free networks in the same limit it is 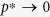. Note also that a bond percolation process can be mapped into a SIR model with infection rate *β* and uniform infection time *τ*. In fact, by defining the trasmissibility 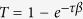 as the probability that the infection will be transmitted from an infected node to at least a susceptible neighbor before recovery takes place, the set of nodes reached by a SIR epidemic outbreak originated from a single node is statistically equivalent to the cluster of the bond percolation problem (with 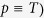 the initial node belongs to^36^.

We then move to properties defined for directed graphs:

- Link reciprocity *r*, measuring the tendency of node pairs to form mutual connections. It is defined as the ratio between the number of bidirected links and the total number of network connections: 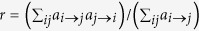. Reciprocity is considered a sensible parameter for systemic risk, giving a measure of direct mutual exposure among nodes.
- Average shortest path length *λ*^22^, where the shortest path length 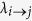 from node *i* to node *j* is the minimum number of links required to connect *i* to *j* (following link directions), and 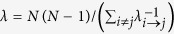 (the harmonic mean is commonly used to avoid problems caused by pairs of nodes that are not reachable from one to another, and for which *λ* diverges). This quantity measures the number of steps that are required, on average, for a signal or a shock to propagate between any two nodes of the network.
- The Group DebtRank *DR*^4^, a measure of the total economic value in the network that is potentially affected by a distress on all nodes amounting to 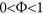, with 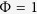 meaning default. In a nutshell, *DR* is based on computing the recursive impact (*i.e.*, the reverberation on the network) of the initial distress, and is defined as:

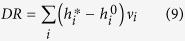


where 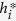 is the final amount of distress on *i* 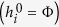 and 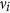 is the relative economic value of *i*. We refer to the original paper^4^ for the details on how to compute *DR*, recalling here that *DR* builds upon the detailed information on individual link weights in the network.

## Results

### Test of FiCM modeling

When testing our network reconstruction procedure it is important to keep in mind that the method is subject to three different kind of errors. The first one comes from hypothesis I that the real network *G*_0_ can be properly described by a CM, whose Lagrange multipliers are obtained by constraining the whole in-degree and out-degree sequences^13^. The second one derives instead from hypothesis II that the node fitnesses 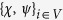 are proportional to the CM’s Lagrange multipliers 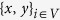, *i.e*., from imposing a FiCM. Finally, the third one is due to the limited information available for calibrating the FiCM and obtain the true value of *z*—namely, the partial knowledge of the in-degree and out-degree sequences. Note however that the first source of mistakes cannot be controlled for in our context, as finding the CM that describes the data requires the knowledge of the whole in-degree and out-degree sequences (which is not accessible for our case studies). This is exactly why we have to make hypothesis II and impose a FiCM by assigning *ad hoc* values to the Lagrange multipliers. In this section we thus concentrate on the second source of errors.

**Figure 3:**
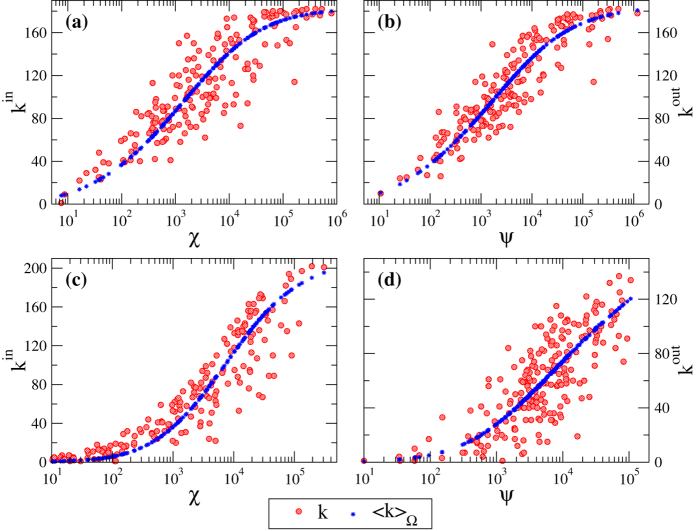
Qualitative assessment for the FiCM description of the real network *G*_0_. Scatter plots of node fitnesses 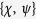 versus real node in- and out-degrees 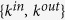 of *G*_0_ (red circles) and their ensemble averages computed via the FiCM (blue asterisks). Upper panels (**a**,**b**) refer to WTW, lower panels (**c**,**d**) to E-mid.

Indeed, real networks are not perfect realizations of the FiCM and can only be approximated by it^12^. In order to assess qualitatively how well this FiCM describes the real network *G*_0_, one can compare the observed in-degrees and out-degrees of *G*_0_ with their averages 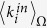 and 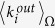 computed on the FiCM ensemble Ω. Figure 3 shows such comparison when the average degrees are obtained through eq. (3) for a fully informed FiCM, *i.e*., with the value of *z* computed via eq. (4) using the knowledge of in- and out-degrees for all nodes. We indeed observe a remarkable agreement between these quantities for our empirical networks: the real degrees are scattered around the functional form of their expected values. The amount of deviations from perfect correlation (which would correspond to an actual realization of the FiCM) gives an indication of how well our model describes the real network. Note that the validity of hypothesis II can be evaluated also in the case of partial information by performing such comparison on the subset *I* of nodes whose topological properties are available.

**Figure 4:**
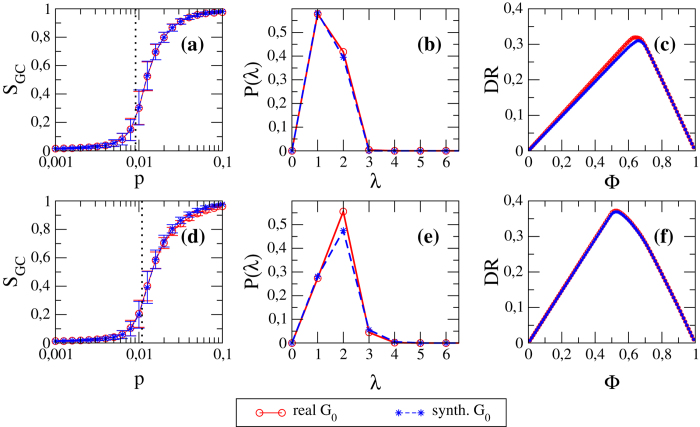
Properties of real and synthetic networks. Left plots (**a**,**d**): dependence of the size of the giant component *S*_*GC*_ on the occupation probability *p* (the vertical dotted line indicates p*). Central plots (**b**,**e**): probability distribution of the directed shortest path length *λ*. Right plots (**c**,**f**): dependence of *DR* on the initial distress Φ. Top panels (**a**–**c**) refer to WTW, bottom panels (**d–f**) to E-mid. Correlation coefficient values *c* between real and synthetic curves: (**a**) *c* = 0.999, (**b**) *c* = 0.999, (**c**) *c* = 0.994, (**d**) *c* = 0.999, (**e**) *c* = 0.989, **(f**) *c* = 0.998.

In the following, in order to have a quantitative global assessments of the errors caused by hypothesis II, we will test our network reconstruction method both on real networks and on benchmark synthetic networks numerically generated with the fully informed FiCM through eq. (3). In the latter case, the errors made by the method will be due only to the limited information available about the degree sequences. It is then interesting to check whether such generated synthetic networks are equivalent to the real networks in term of systemic risk. Figure 4 shows that bond percolation properties, shortest path length distribution and DebtRank values of synthetic networks are in excellent agreement with those of their real counterparts (the correlation coefficients between real and synthetic curves are all above 0.99). FiCM thus proves itself to be a proper framework for modeling our empirical networks.

### Test against limited information

In this section we finally proceed to the key testing of the method against the third (and more relevant) source of errors: the limitedness of the information available on the degree sequences for calibrating the FiCM. In order to obtain a quantitative estimation of the method’s effectiveness in reconstructing a topological property *X* of a given a network *G*_0_ (which can be either the real one or its synthetic version), we implement a procedure consisting in the following operative steps:

- Choose a value of *n* < *N* (the number of nodes for which the in- and out- degrees are known).
- Build a set of *M* = 100 subsets 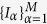 of *n* nodes picked at random from *G*_0_.
- For each subset *I*_*α*_, use the degree sequences from *G*_0_ to evaluate *z* from eq. (4), and name such value *z*_*α*_.
- Build the ensemble 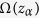 using the linking probabilities from eq. (3): generate *m* = 100 networks from Ω(*z*_*α*_), and compute the average value *X*_*α*_ of property *X* on this ensemble.
- Compute the relative root mean square error (rRMSE) of property *X* over the subsets 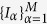:

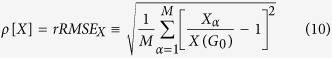


where 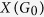 is the value of *X* measured on *G*_0_.

**Figure 5:**
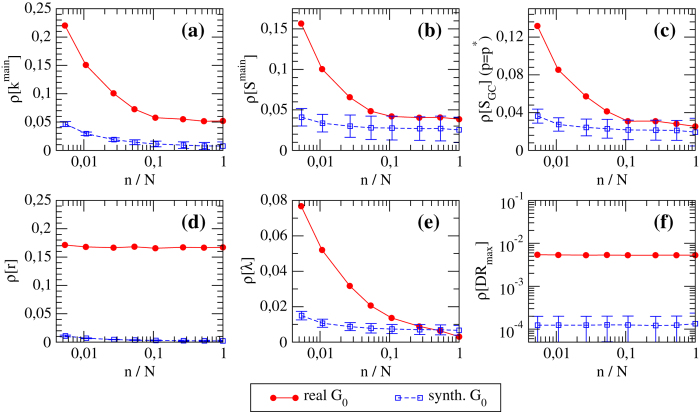
rRMSE of various topological properties versus *n* for the reconstructed *G*_0_ of the WTW (both the real network and its synthetic version). rRMSE for: (**a**) degree of the main core *k*^*main*^, (**b**) size of the main core *S*^*main*^, (**c**) size of the giant component *S*_*GC*_ at the bond percolation threshold 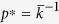, (**d**) link reciprocity *r*, (**e**) mean shortest path length *λ*, (**f**) maximum value of the group DebtRank *DR*_*max*_.

**Figure 6:**
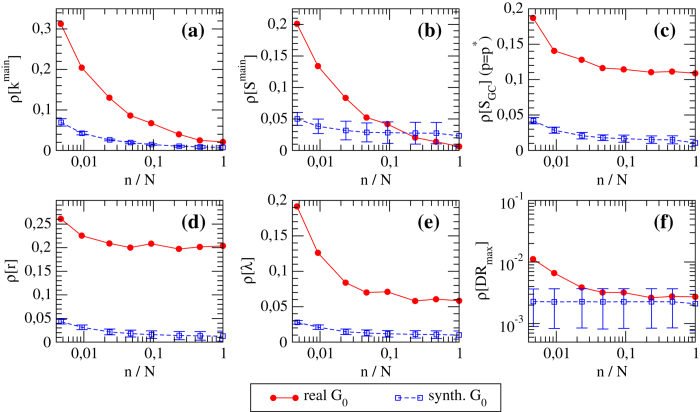
rRMSE of various topological properties versus *n* for the reconstructed *G*_0_ of the E-mid (both the real network and its synthetic version). rRMSE for: (**a**) degree of the main core *k*^*main*^, (**b**) size of the main core *S*^*main*^, (**c**) size of the giant component *S*_*GC*_ at the bond percolation threshold 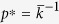, (**d**) link reciprocity *r*, (**e**) mean shortest path length *λ*, (**f**) maximum value of the group DebtRank *DR*_*max*_.

We then study how the rRMSE for the various network properties we consider varies as a function of the size *n* of the subset of nodes used to calibrate the FiCM (*i.e*., for which in- and out- degree information is available). Results are shown in Figs 5 and 6. We observe that in most of the cases there is a rapid decrease of the relative error as the number of nodes *n* used to reconstruct the topology increases. For instance, generally the error drops to half of the starting rRMSE (for *n* = 1) at *n*/*N* = 5%, and to one quarter for 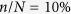
—a value that is rather close to that of the final error made at 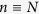. This is an indication of the goodness of the estimation provided by our method. As expected, the rRMSE is higher for real networks than for synthetic networks, and the difference between the two curves gives a quantitative estimation of the error made in modeling real networks with the FiCM. The fact that such a difference is higher for E-mid than for WTW is directly related to a slightly better correlation between real and expected degrees observed in the latter case (Fig. 3). Note that the various rRMSE for synthetic networks do not necessarily tend to zero, because the generated synthetic configuration might be highly improbable—in some cases, the synthetic network can be even more atypical than the real one. We thus indicate with error bars the range of performance of our method for different choices of synthetic *G*_0_.

Generally, *S*_*GC*_, *λ*, *k*_*main*_ and *S*_*main*_ are the properties which are reconstructed better: for instance, with the knowledge of only 10% of the nodes, all the relative errors become smaller than 10%, and they decrease for increasing *n*. The rRMSE for *r* and *DR* show instead a behavior almost flat in *n*. The fact that the rRMSE for *r* computed for real networks remains steadily high is probably due to the fact that reciprocity is hardly reproduced by a directed CM, and is better suited as additional imposed constraint^26^. The rRMSE for *DR* is instead remarkably small for real networks (with values around 0.5%), and we can thus conclude that our method is efficient in estimating *DR* also when the available information is minimal. This is particularly relevant to our analysis, since we are estimating *DR* at its peak (*i.e*., at its maximum, and thus mostly fluctuating, value), where the details of the weighted topology play a fundamental role in the process of risk propagation. Besides, and more importantly, the value of *DR* for the real network is computed using the original weighted topology, whereas, the computation of *DR* in the reconstructed network builds on link weights obtained by the degree-corrected gravity prescription of eq. (7).

In conclusion, the outcome of this analysis is that our network reconstruction method is able to estimate the network properties related to systemic risk with good approximation, by using the information on the number of connections of a relatively small fraction of nodes—as long as the fitnesses of all nodes is known.

## Discussion

In this paper we studied a novel method that allows to reconstruct a directed weighted network and estimate its topological properties by using only partial information about its connection patterns, as well as two additional intrinsic properties (interpreted as fitnesses) associated to each node. Tests on empirical networks as well as on synthetic networks generated through a fitness-induced configuration model reveal that the method is highly valuable for overcoming the lack of topological information that often hinders the estimation of systemic risk in economic and financial systems. Indeed, the information exploited by the method is minimal but is (or should be) publicly available for these kind of systems.

Our work originates from the study of Garlaschelli and Loffredo^12^ and of Musmeci *et al*.^10^. The latter in particular represented a first attempt in tackling the problem of network reconstruction from partial information within the framework of fitness-induced configuration models. Here however we make fundamental improvements to the method, the key advance being that of extending it to directed weighted networks (the most general class of networks). In the present form, the method is then suited to reconstruct high-order network properties related to systemic risk, a task of primary practical importance the method was conceived to address—that was however beyond the reach of its original version. Besides, the validation of the fitness-induced configuration model approach to model real networks, as well as the reconstruction of benchmark synthetic networks generated as fitness-based counterparts of the empirical networks, are both novel ingredients that allow to assess quantitatively the accuracy of the method. Last but not least, the extensive analysis of different temporal snapshots of the real networks we provide in the Supplementary Information allows to strengthen considerably the effectiveness and robustness of our method.

We remark that the method we are proposing here, by reproducing both the binary and weighted topology of the network, represent a substantial step forward in the field of network reconstruction. In fact, most of the previous works^6^,^7^,^8^,^9^,^25^ focused on reproducing the strengths of the real network to the detriment of connection patterns, whereas, only recently it has been realized that a successful reconstruction procedure must resort also on topological constraints^2^,^10^. Here we are proposing a method that allows to always reproduce the strengths, but also to tune the network topology through appropriate connection probabilities. In this respect, the use of probabilities derived from degree constraints represent the most general case, which include as specific instances both the dense reconstruction^6^,^7^,^8^,^9^ and the sparse reconstruction^2^ techniques.

Note that one should not be much surprised that the knowledge of a small number of nodes allows to precisely estimate a wide range of network properties, because the method assumes the additional knowledge of the fitness parameters for all the nodes. Besides, the effectiveness of the method strongly depends on the accuracy of the fitness model used to calibrate the CM in order to fit the empirical dataset. In the case of WTW and E-mid, the fitness model well describes how links are established across nodes, and our method is thus effective in reconstructing the network properties. Finally, we remark that the issue of having limited information on the system under investigation, while being typical for social, economic and financial systems (that are privacy-protected), is very relevant also for biological systems such as ecological networks, metabolic networks and functional brain networks—where, due to observational limitations and high experimental costs for collecting data, detailed topological information about connections is often missing. Notably, our method can be used to reconstruct any network representing a set of (directed and weighted) dependencies among the constituents of a complex system, and we thus believe it will find wide applicability in the field of complex networks and statistical physics of networks.

## Additional Information

**How to cite this article**: Cimini, G. *et al*. Systemic Risk Analysis on Reconstructed Economic and Financial Networks. *Sci. Rep*. **5**, 15758; doi: 10.1038/srep15758 (2015).

## Supplementary Material

Supplementary Information
