# Supplementary material for: Systemic Risk Analysis on Reconstructed Economic and Financial Networks
Source: Sci Rep. 2015 Oct 28;5:15758. doi: 10.1038/srep15758 (PMC4623768; doi:10.1038/srep15758)
Supplement: Supplementary Information [file srep15758-s1.pdf]

Systemic risk analysis on reconstructed economic and financial networks

Giulio Cimini,[1] Tiziano Squartini,[1] Diego Garlaschelli,[2] and Andrea Gabrielli[1,3]

[1] *Istituto dei Sistemi Complessi (ISC-CNR) UoS "Sapienza" Università di Roma, 00185 Rome, Italy*
[2] *Lorentz Institute for Theoretical Physics, University of Leiden, 9506 Leiden, Netherlands*
[3] *IMT Institute for Advanced Studies, 55100 Lucca, Italy*

SUPPLEMENTARY INFORMATION

Here we provide the results of our analysis for several temporal snapshots of the dataset we consider, namely the World Trade Web (WTW) and the Electronic Market for Interbank Deposits (E-mid). In particular, concerning the WTW, we have analyzed the whole database [1] that covers a period of 51 years (1950-2000); here we show only a snapshot every five years since this subsample is informative enough to provide a faithful representation of the WTW evolution. Concerning E-mid instead, the database [2] covers a period of 14 years (1999-2012); here we shown all yearly snapshots, since substantial structural changes of this network occur at a much faster rate than those of the WTW.

Figures S1 and S2 (which correspond to Figure 3 of the main text) show that the FiCM (fitness-induced configuration model) well approximates the real networks G_0, as the observed in-degrees and out-degrees of G_0 are scattered around their averages $\langle k_i^{in}\rangle_\Omega$ and $\langle k_i^{out}\rangle_\Omega$ computed on the FiCM ensemble Ω. Figures S3 and S4 (which correspond to Figure 4 of the main text) show instead that the properties of synthetic networks generated through the FiCM are equivalent to those of their real counterparts, giving additional support to our hypothesis that the FiCM is a proper framework to model our empirical networks.

Finally we move to the analysis of the rRMSE for the various network properties we consider, focusing on how it varies as a function of the size n of the subset of nodes used to calibrate the FiCM. Figures S5 and S6 (which correspond to Figure 5 of the main text) report the results for WTW snapshots, which confirm most of the observation made in the main text. Indeed, λ, S_{GC}, k_{main} and S_{main} are the properties which are reconstructed better: for instance, with the knowledge of only 5%-10% of the nodes, the relative errors are smaller than 4%, 9%, 13% and 16%, respectively. The rRMSE for r shows instead a behavior almost flat in n, and its value ranges from 10% to 20%. Finally, the rRMSE for DR is also almost flat in n, and remarkably it is always smaller than 1%.

Figures S7 and S8 (which correspond to Figure 6 of the main text) report instead the results for E-mid snapshots. We observe here that the analysis is less stable than in the case of WTW, supposedly because the E-mid network is inherently more dynamic, and our time span covers highly unstable regions given by the 2007-2009 financial crisis. Indeed, we can still assert that λ, S_{GC}, k_{main} and S_{main} are the properties which are reconstructed better, as their relative errors generally range from 5% to 15% when knowing only 5%-10% of the nodes. However, such errors increase substantially during years 2008-2010, when also the rRMSE for r grows up to 60% (from original values of about 30%). The DR is instead (and remarkably) generally reconstructed with high accuracy. Note that the rRMSE for both r and DR shows a monotonic increase in time (the latter in particular grows of a factor 10 from 0.5% in 1999 to 5% in 2010, which is also probably related to the increase of the DR maximum value—see Figures S3 and S4), whereas, for S_{main} we observe a large rRMSEs also during years 2002-2005 (*i.e.*, starting from the "Internet bubble"). Summing up, financial crises appear to have an effect of making the network less "random"—a fact that makes the FiCM less suited as modeling framework. This, however, can in turn be seen as an indicator of financial instability, which is an important result itself that deserves further attention.

[1] http://privatewww.essex.ac.uk/ ksg/exptradegdp.html
[2] http://www.e-mid.it/data-service-en/68-e-mid-for-research/197-data-providing.html

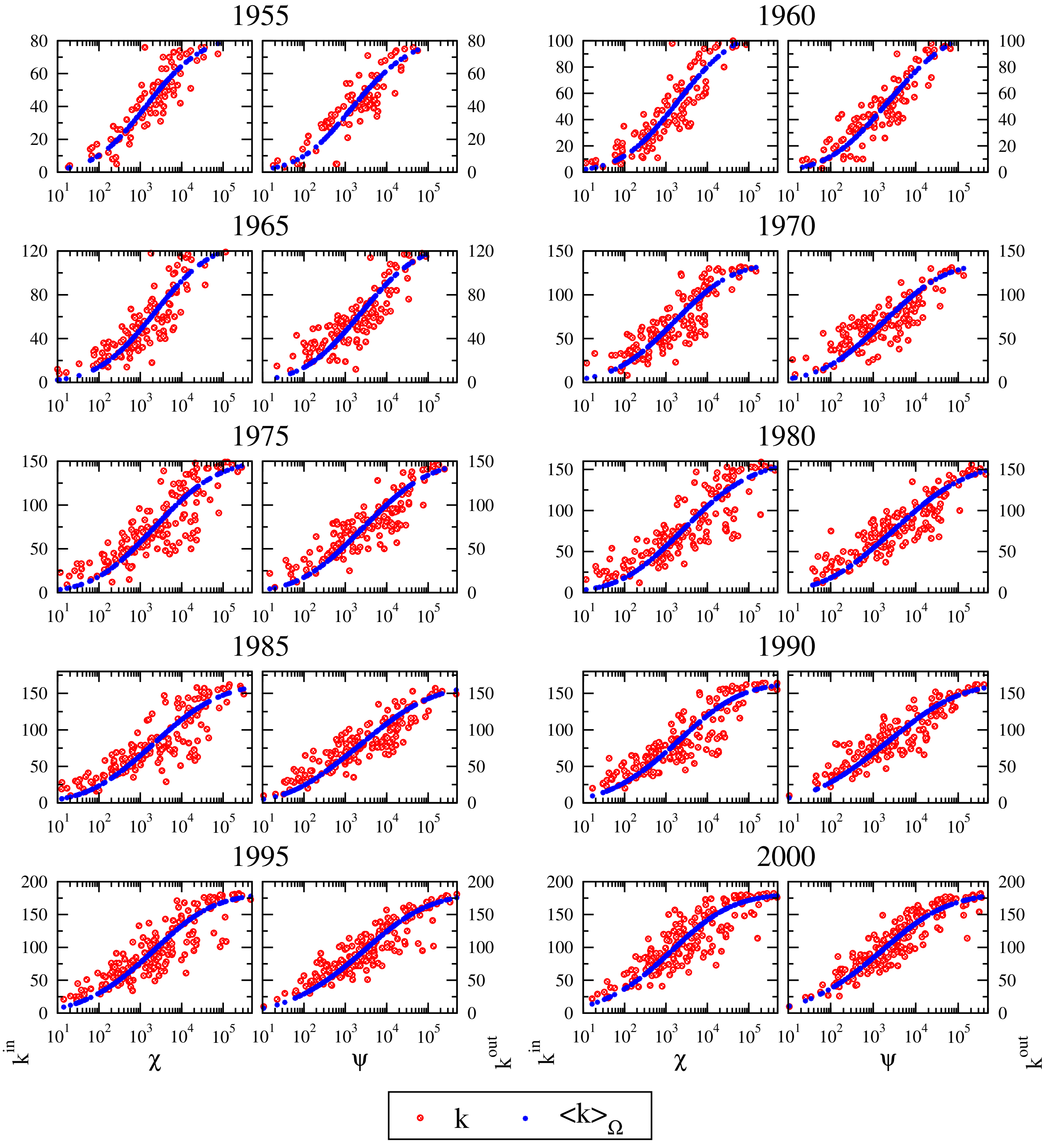

FIG. S1. WTW, various yearly snapshots: Scatter plots of node fitnesses $\{\chi, \psi\}$ versus real node in- and out-degrees $\{k^{in}, k^{out}\}$ of G_0 (red circles) and their ensemble averages computed via the FiCM (blue asterisks). Refer to last row of plots for axes labels.

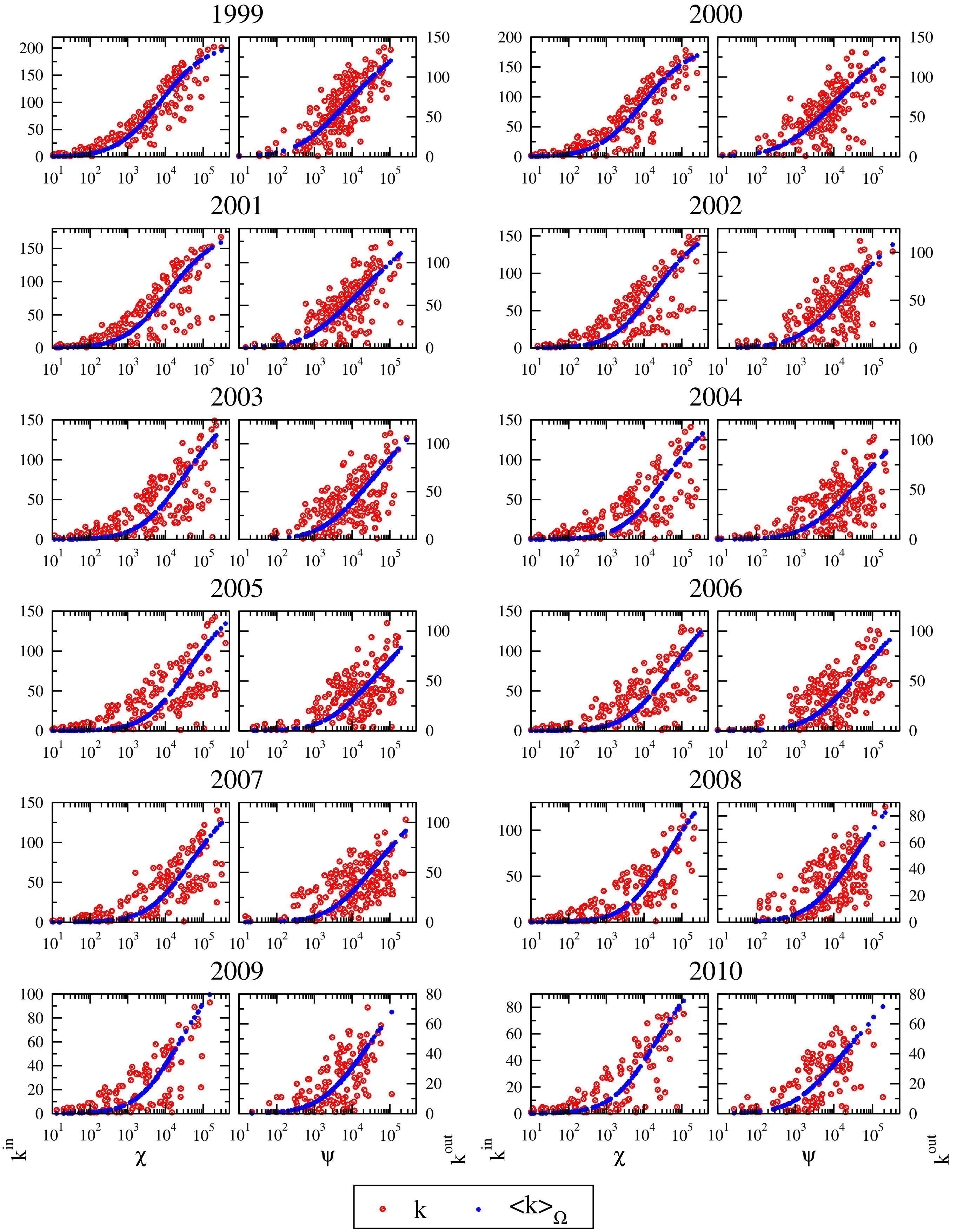

FIG. S2. E-mid, various yearly snapshots: Scatter plots of node fitnesses $\{\chi, \psi\}$ versus real node in- and out-degrees $\{k^{in}, k^{out}\}$ of G_0 (red circles) and their ensemble averages computed via the FiCM (blue asterisks). Refer to last row of plots for axes labels.

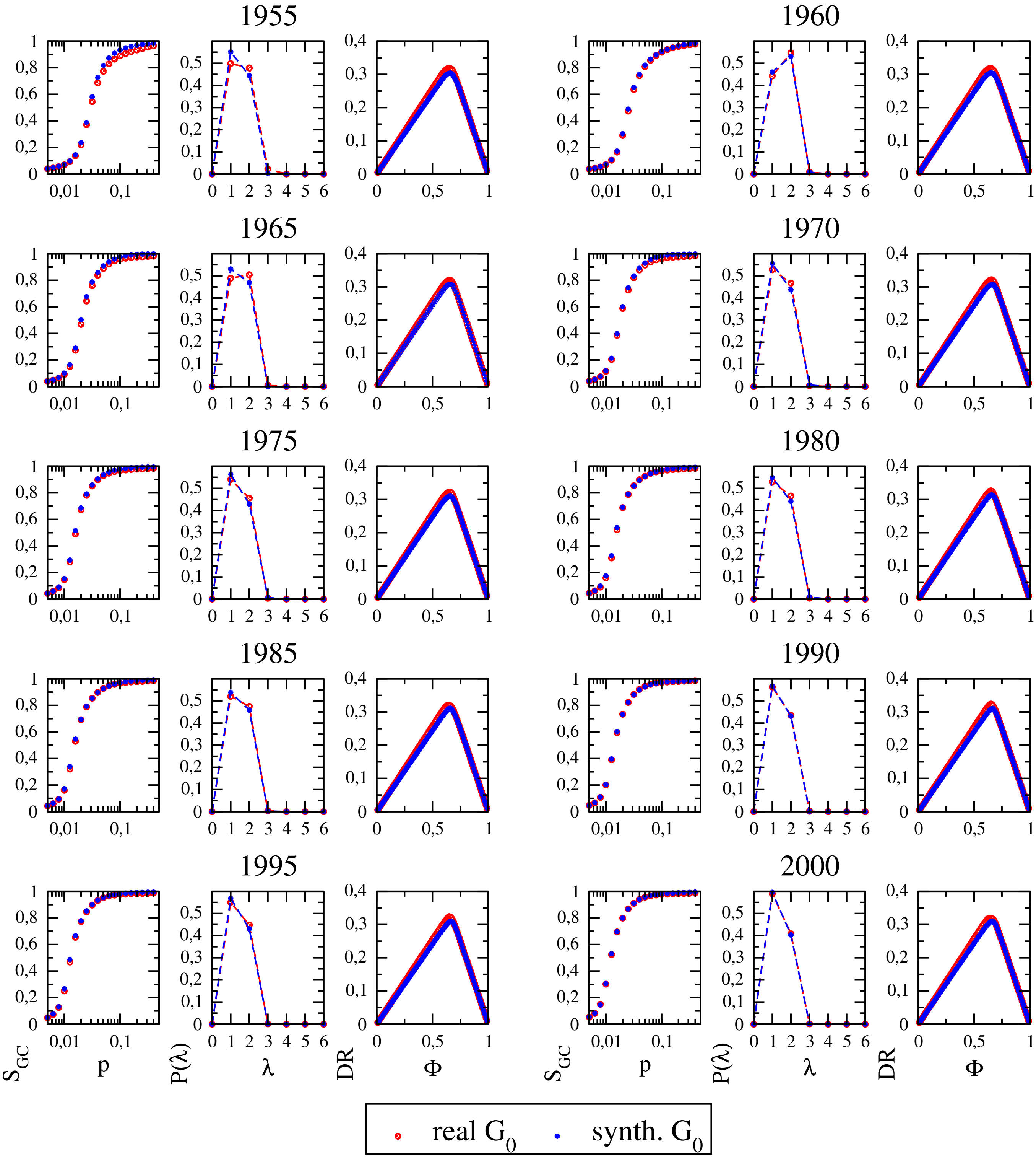

FIG. S3. WTW, various yearly snapshots: (left) dependence of the size of the giant component S_{GC} on the occupation probability p; (center) probability distribution of the directed shortest path length λ; (right) dependence of DR on the initial distress Φ. Refer to the last row of plots for axes labels.

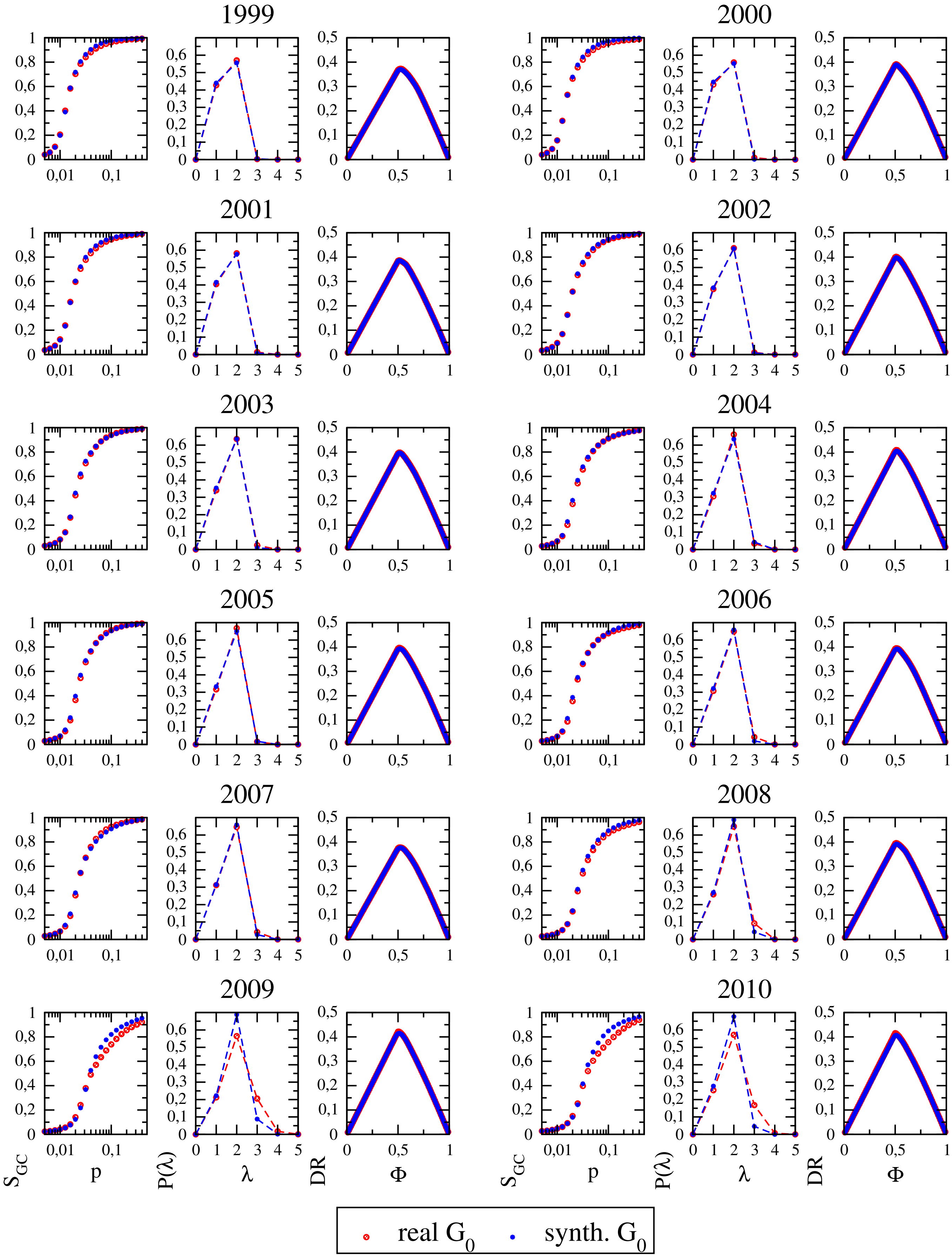

FIG. S4. E-mid, various yearly snapshots: (left) dependence of the size of the giant component S_{GC} on the occupation probability p; (center) probability distribution of the directed shortest path length λ; (right) dependence of DR on the initial distress Φ. Refer to the last row of plots for axes labels.

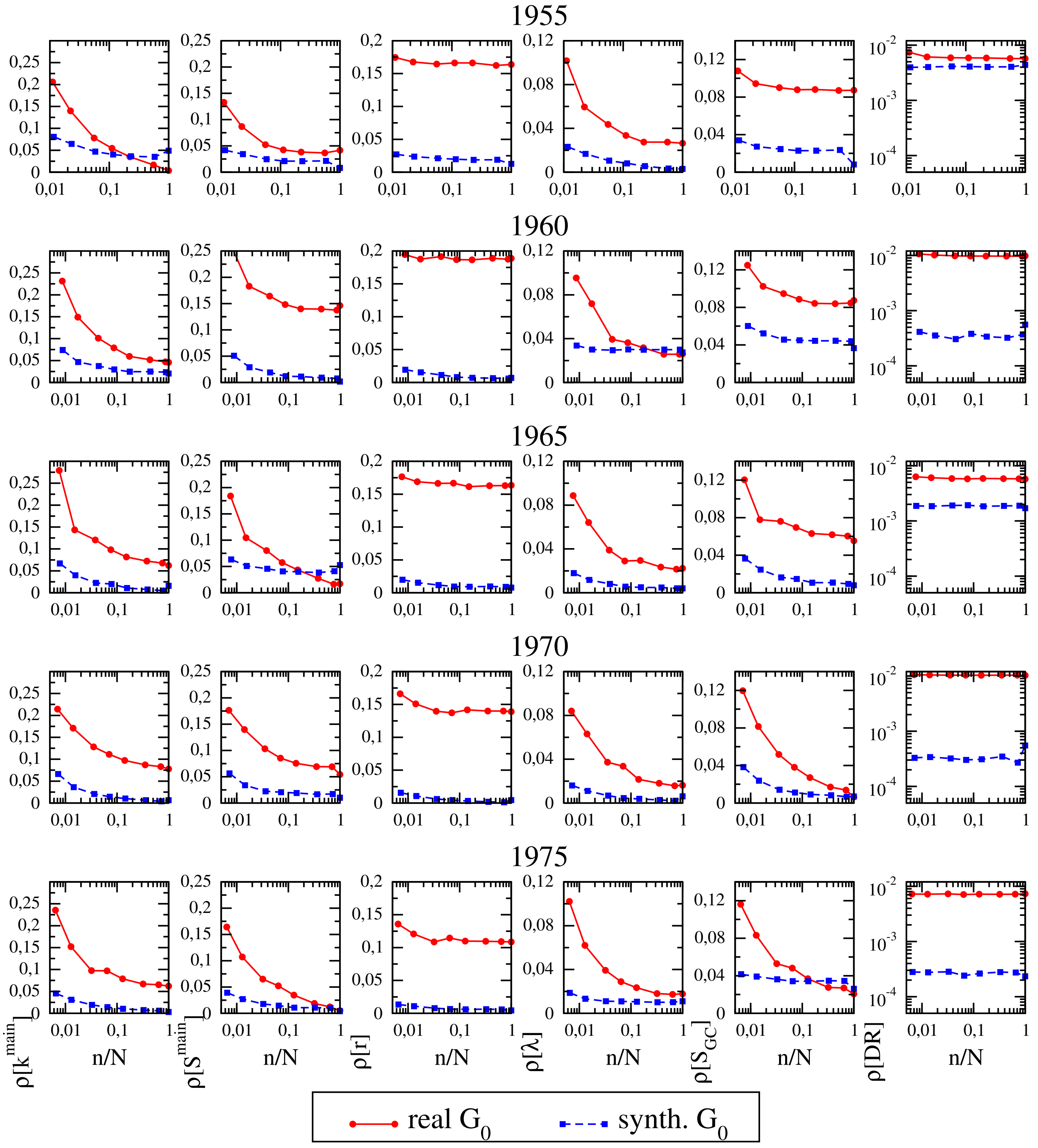

FIG. S5. WTW, various yearly snapshots (part 1): rRMSE versus n of the various topological properties for the reconstructed G_0 (both the real network and its synthetic version). Refer to the last row of plots for axes labels—each column of plots corresponds to a different quantity.

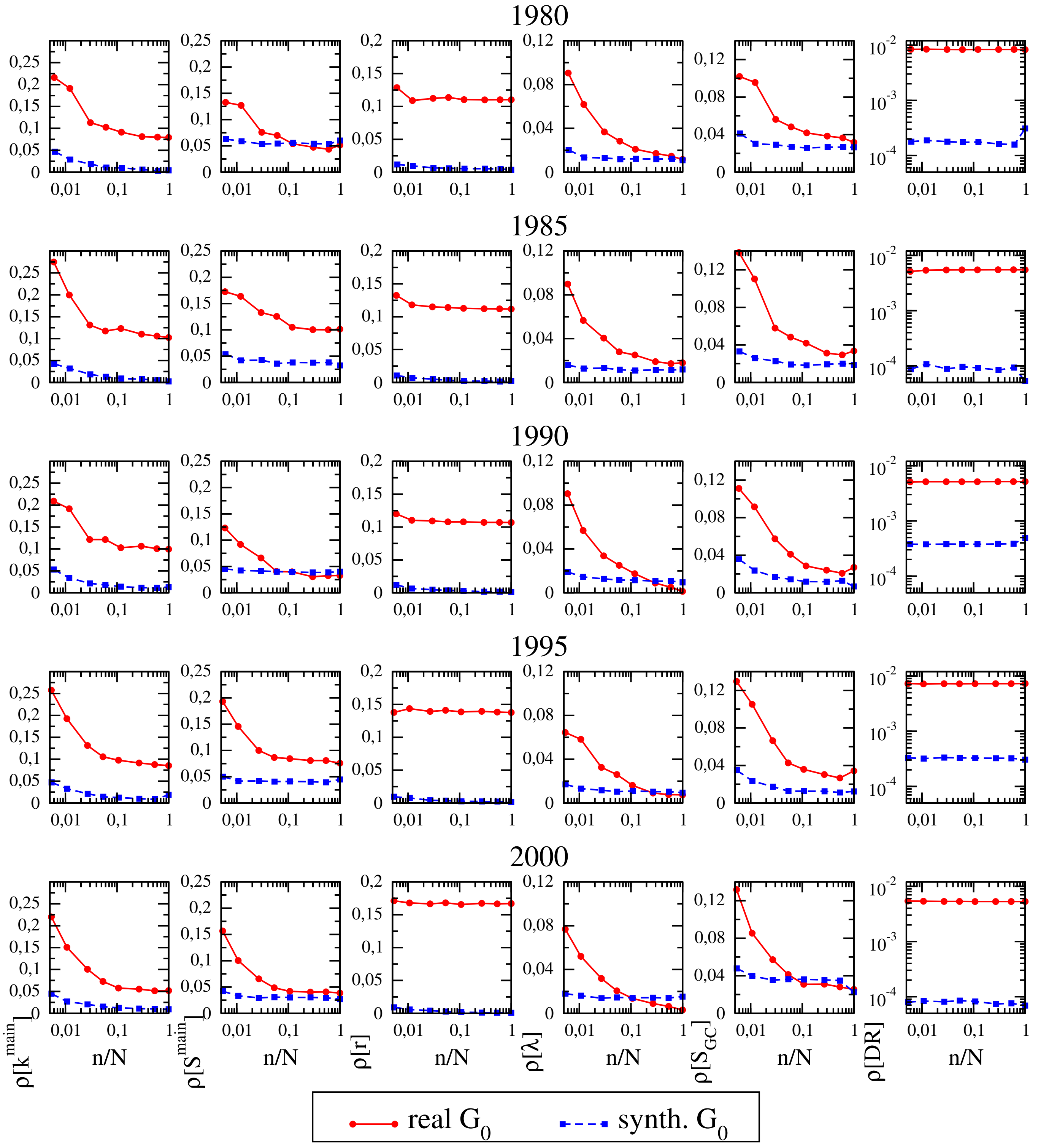

FIG. S6. WTW, various yearly snapshots (part 2): rRMSE versus n of the various topological properties for the reconstructed G_0 (both the real network and its synthetic version). Refer to the last row of plots for axes labels—each column of plots corresponds to a different quantity.

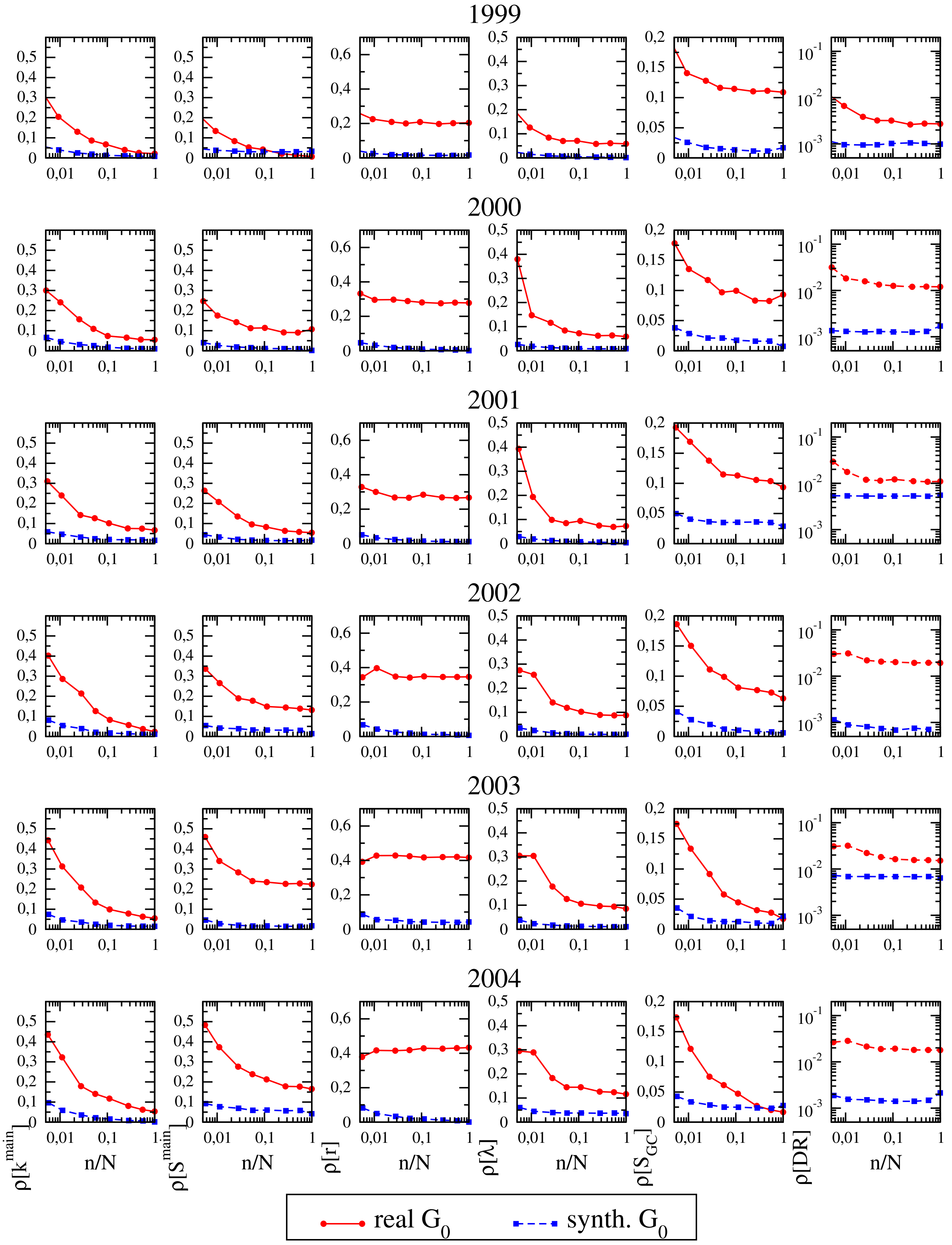

FIG. S7. E-mid, various yearly snapshots (part 1): rRMSE versus n of the various topological properties for the reconstructed G_0 (both the real network and its synthetic version). Refer to the last row of plots for axes labels—each column of plots corresponds to a different quantity.

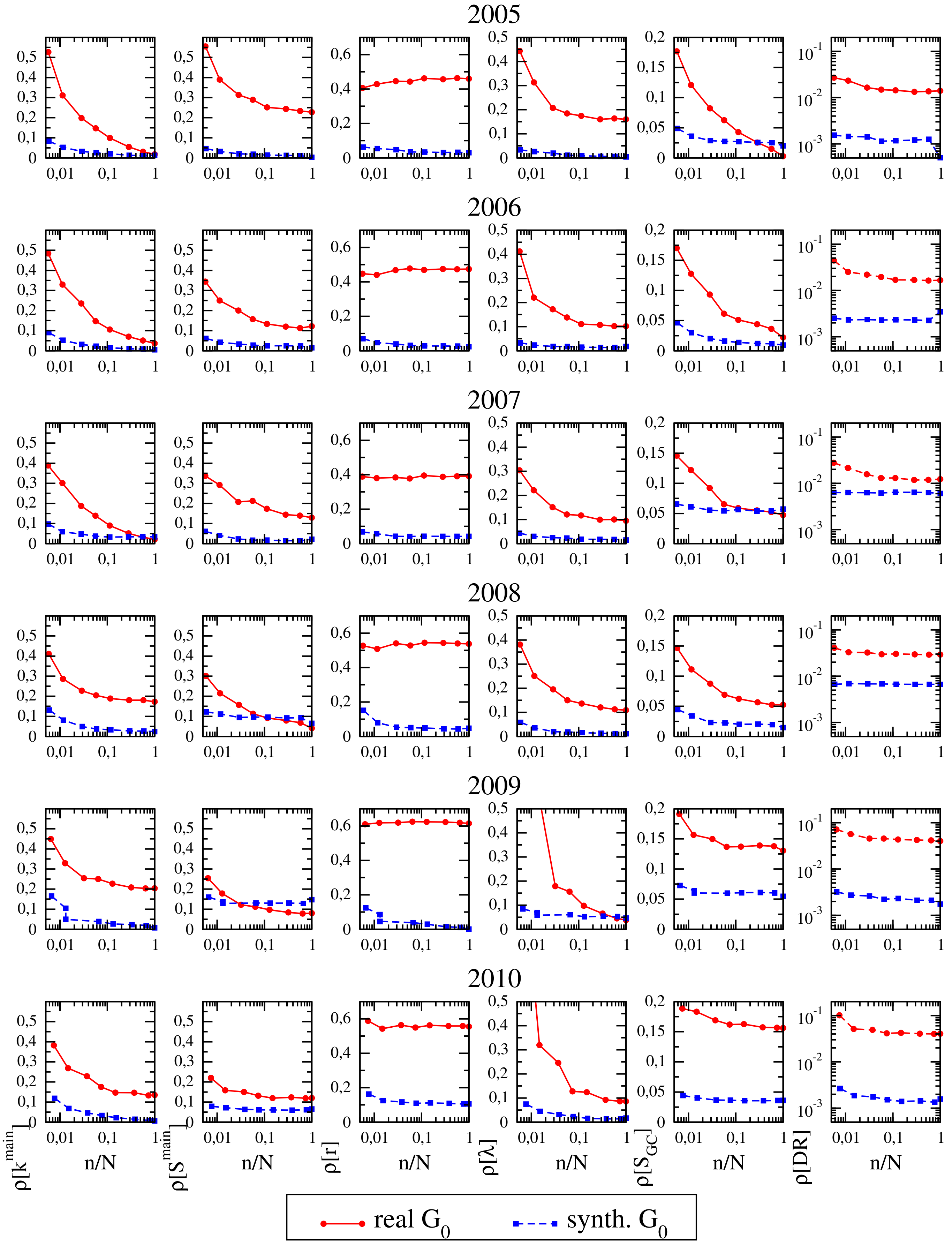

FIG. S8. E-mid, various yearly snapshots (part 2): rRMSE versus n of the various topological properties for the reconstructed G_0 (both the real network and its synthetic version). Refer to the last row of plots for axes labels—each column of plots corresponds to a different quantity.
